# The Role of the Three Rs in Improving the Planning and Reproducibility of Animal Experiments

**DOI:** 10.3390/ani9110975

**Published:** 2019-11-14

**Authors:** Adrian J. Smith, Elliot Lilley

**Affiliations:** 1 Norecopa, c/o Norwegian Veterinary Institute, P.O. Box 750 Sentrum, 0106 Oslo, Norway; 2 Science Group, Research Animals Department, RSPCA, Wilberforce Way, Southwater, West Sussex RH13 9RS, UK; elliot.lilley@rspca.org.uk

**Keywords:** Three Rs, planning, reproducibility, prepare, reporting

## Abstract

**Simple Summary:**

Efforts to improve the design of animal studies have tended to focus on the more mathematical aspects, such as randomization, blocking and statistical analysis. There are, however, many other factors that affect the data from preclinical studies. To improve validity, scientists must collaborate closely with the animal facility involved as soon as the decision to use animals has been made. Such discussions will also help to improve animal welfare, as well as any health and safety issues. A large number of guidelines have been produced over the last 20 years for *reporting* results in the scientific literature. Comprehensive guidelines for *planning* animal experiments have been produced more recently, to fulfill this need. These will be described in this paper. A commitment to improving animal welfare, scientific quality, staff care and transparency for all stakeholders will also foster a culture of care around animal research, which benefits all parties. All the Three Rs of Russell and Burch (Replacement, Reduction and Refinement) play a role in the planning and reproducibility of research and testing which may involve animals.

**Abstract:**

Training in the design of animal experiments focuses all too often on those aspects which can be approached mathematically, such as the number of animals needed to deliver a robust result, allocation of group size, and techniques such as randomization, blocking and statistical analysis. Important as they are, these are only a small part of the process of planning animal experiments. Additional key elements include refinements of housing, husbandry and procedures, health and safety, and attention at all stages to animal welfare. Advances in technology and laboratory animal science have led to improvements in care and husbandry, better provision of anesthetics and analgesics, refined methods of drug administration, greater competence in welfare assessment and application of humane endpoints. These improvements require continual dialogue between scientists, facility managers and technical staff, a practice that is a key feature of what has become known as the culture of care. This embodies a commitment to improving animal welfare, scientific quality, staff care and transparency for all stakeholders. Attention to both the physical and mental health of all those directly or indirectly involved in animal research is now an important part of the process of planning and conducting animal experiments. Efforts during the last 30 years to increase the internal and external validity of animal experiments have tended to concentrate on the production of guidelines to improve the quality of *reporting* animal experiments, rather than for *planning* them. Recently, comprehensive guidelines for *planning* animal studies have been published, to redress this imbalance. These will be described in this paper. Endorsement of this overarching influence of the Three R concept, by all the stakeholders, will not only reduce animal numbers and improve animal welfare, but also lead to more reliable and reproducible research which should improve translation of pre-clinical studies into tangible clinical benefit.

## 1. Introduction

In the 1950s, when Russell and Burch were tasked by the Universities Federation for Animal Welfare (UFAW) to investigate the status of animal experimentation in the UK [1], opposition to animal research was based mainly upon its perceived inhumanity. Alternatives to animal models were in their infancy, and the concept of Three Rs (Replacement, Reduction, Refinement) which they developed focused therefore naturally on ways of reducing inhumanity, hence the title of their book: The Principles of Humane Experimental Technique [2].

Today, animal research is receiving challenges from a new and unexpected quarter: a group of eloquent scientists who are pointing out serious flaws in the design and reporting of many preclinical studies. These flaws raise concerns about the ability of laboratory animal models to represent the target population (usually humans), for example in the case of Alzheimer’s disease [3], resulting in poor translational success rates for many important human conditions, such as stroke, multiple sclerosis, Parkinson’s disease, inflammatory bowel disease and cancer [4,5]. The Three Rs of Russell and Burch have been joined by the Three Vs of Hanno Würbel: construct validity, internal validity and external validity [6]. Words such as ‘false’ [7] and ‘waste’ [8,9] have been used to describe the quality of animal research, which is going through a ‘reproducibility crisis’ [10].

- The major causes of concern raised by these authors relate to operational issues:
- Publication bias;
- Low statistical power;
- P-value hacking;
- HARKING (Hypothesizing After the Results are Known).

These have been dubbed the ‘Four Horsemen of Irreproducibility’ [11]. To these we may add a long list of animal-related items, such as:

- Insufficient information regarding the source, health status, strain, sex and age of the animals;
- Lack of detail with regard to housing, husbandry and care;
- Weaknesses regarding definition of the experimental unit;
- Artifacts caused by procedures or extraneous environmental factors;
- Suspicions of inadequate post-operative analgesia;
- Poor compliance with guidance for reporting animal experiments.
- A collection of literature references to these concerns is available on Norecopa’s website [12].

Some of these errors, such as publication bias (the tendency for only papers reporting positive findings to be published [13]) are clearly not only the responsibility of the scientists themselves. The wider scientific community, including funding bodies and journals, must encourage the publication of both negative results and replication studies. Greater use of pre-registration sites will help in this respect [14,15,16,17]. The culture of ‘publish or perish’ that encourages publication quantity over quality, and overzealous focus on high-impact journals, also needs to be challenged. This requires changes in attitudes at many levels, including more time spent on testing the reproducibility of experimental findings in order to evaluate their robustness and generalizability [18], as well investigating the possibility of redirecting funds to non-animal methods.

The majority of the papers highlighting this ‘reproducibility crisis’ focus on weaknesses or errors in the creation and use of treatment and control groups, and in the statistical analyses of the data from them. Papers offering solutions to the current situation (e.g., [17]) tend therefore to focus on issues which can be approached mathematically, such as the number of animals needed to deliver robust data, determination of group size, techniques such as randomization, blocking and blinding, and methods for analysis of the data, to avoid HARKING [19] and p-hacking [20]. Many of the tools for improving planning, such as the Experimental Design Assistant [21], are therefore also based on these elements. While robust experimental design is important, those working within, or in close contact with, animal facilities recognize a host of other factors which are equally important. Indeed, we doubt that Russell and Burch would have been satisfied by a purely mathematical approach to the design of animal experiments. Emphasizing experimental design over all of the other elements that contribute to an animal study fails not only to address the R of Replacement, but also the many efforts being made to Reduce the numbers of sentient animals by other means, and to Refine studies to reduce pain, suffering or lasting harm. It is now widely accepted that improvements in animal welfare have the added benefit of producing better science [22,23]. In this connection, a themed issue of the ILAR Journal in 2014 on experimental design and statistics contained a healthy mix of papers, not only on the mathematics of study design but also on animal-related issues [24].

## 2. Reporting Cannot Solve the Reproducibility Issue Alone 

Until recently, efforts to resolve the ‘reproducibility crisis’ with respect to animal experiments have focused on guidelines to improve the quality of their reporting in the scientific literature. Improvements have certainly been needed, as Jane Smith and colleagues pointed out over 20 years ago [25] in an analysis of 149 papers from 8 journals, where, for example, over 50% of papers failed to report the age, weight or source of the animals, and 30% neglected to mention the number used. A similar study later showed how valuable space is often wasted by using meaningless expressions such as ‘farm pigs’ (instead of mentioning the breed, source, sex and age), or ‘recommended conditions’ (with no indication as to who has recommended them) [26]. Jane Smith’s survey also revealed an unsettling use of phrases such as ‘tools’ and ‘preparations’ for animal models, which can lead to a less empathetic view of the animals, as well as damaging the public perception of animal research. There is now no shortage of generic, model-specific or journal-related guidelines for reporting animal studies (e.g., [27,28,29,30,31,32]). Disturbingly, compliance with them, even after journal endorsement of these guidelines, has been shown to be unsatisfactory [33,34,35], which in turn can affect reviewers’ attitudes to them. Those who are in the process of updating the reporting guidelines which have been most widely endorsed (ARRIVE), themselves state that the amount of work needed to ensure compliance is unlikely to be sustainable for most journals, because of the resources needed [35].

It should be obvious that an experiment can only be reported successfully if it is planned and carried out equally successfully. Focusing solely on improvement of the reporting of experiments will only, with time, partially deal with the problem of irreproducibility, and it cannot improve experiments which have already taken place - one cannot improve a burnt cake after it has been baked. It is therefore both logical and meaningful to offer scientists *planning* guidelines as well. Indeed, Russell and Burch themselves stated that ‘one general way in which great reduction may occur is by the right choice of *strategies* in the planning and performance of whole lines of research’ [2].

In December 2016 the EU Commission held a conference entitled “The Way Forward”, as part of their response to the European Citizen’s Initiative “Stop Vivisection” which demanded abrogation of Directive 2010/63 and an end to all animal research [36]. During the meeting, participants acknowledged the value of existing reporting guidelines, but recognized that what was needed now were guidelines for *planning* research which might involve animals. This prompted the authors of this paper and colleagues to construct the PREPARE guidelines (Planning Research and Experimental Procedures on Animals: Recommendations for Excellence), which were pre-published in April 2017 and then printed in the August 2018 issue of *Laboratory Animals* [37]. Earlier versions of these guidelines had been in use for many years, in unpublished form, on courses in Laboratory Animal Science at the Norwegian School of Veterinary Science in Oslo.

PREPARE consists largely of statements and knowledge resources which are familiar to managers, veterinarians and other staff at animal facilities. Many of the elements in PREPARE are, however, probably less well-known to those scientists who are not veterinarians, or who are not familiar with the day-to-day management of an animal facility. There are a number of important issues, which can affect both animal welfare and scientific validity, which are not generally highlighted in reporting guidelines. PREPARE consists not only of a 2-page checklist, but, more importantly, comprehensive webpages for each of the 15 topics on the checklist. These pages are updated regularly and provide curated links to global 3R-resources and specific guidelines, on topics such as harm-benefit assessment (e.g., [38]), severity classification (e.g., [39]), drug administration and blood sampling (e.g., [40]), and health monitoring (e.g., [41]). PREPARE also contains links to other checklists for planning specific research models, such as for stroke [42] and osteoarthritis [43].

The PREPARE guidelines aim to be a communication tool. They introduce scientists not only to the large range of topics which can affect the outcome of an experiment, but also to the need for close and early collaboration with staff at the animal facility. This will automatically trigger attention to other important areas such as the need for contingency plans, threat and error management, health and safety issues, and the fate of animals after the study. PREPARE may also awaken a need to consult with specialists in other fields, for example those using non-animal methods. The popularity and impact of the three Rs is surely due to the fact that all stakeholders, not just those with knowledge of design and statistics, can identify with the concept.

The aim of the PREPARE guidelines is not to promote extreme standardization, since this in fact may be counter-productive [44,45,46], nor is it to give scientists more paperwork. PREPARE should be perceived as an *aide memoire* and a source of quality guidelines for all the steps in planning and conducting animal studies. PREPARE is designed to alert scientists to the large range of issues which should be considered at an early stage when planning studies that may require the use of animals. This process of reflection will then ensure that the Three Rs are applied in the order which Russell and Burch originally proposed: Replacement, Reduction, Refinement. If Replacement is impossible, the guidelines will help in the application of Reduction and Refinement. This is similar to the way in which experienced pilots use checklists, even on routine flights: to remember all the steps, to conduct them correctly and in the correct order, and to ensure communication and collaboration with all those involved. *Planning* guidelines have also, naturally, far greater potential than *reporting* guidelines in assisting funders, ethical review committees and regulators in the assessment of applications for new projects [47]. The potential for planning and reporting guidelines, in combination, to promote a greater focus on experimental rigor at all stages of the research cycle has been pointed out by those who have demonstrated that journal endorsement of reporting guidelines has not yet significantly improved the situation [48].

## 3. The Three Rs as a Catalyst for Other Initiatives

### 3.1. The International Culture of Care Network

While Russell and Burch naturally concentrated on ways of reducing inhumanity in animal studies, today’s laboratory animal community has extended this caring attitude to the humans involved as well. The concept of a ‘Culture of Care’ is now widely used to indicate a commitment to improving animal welfare, care of the staff and transparency, involving all stakeholders. Fostering a ‘climate of care’ around animal research is mentioned in recital 31 which precedes the articles in EU Directive 2010/63 [49], and the concept has been endorsed by research bodies in other parts of the world as well.

An International Culture of Care Network has been established to promote this concept, and to share resources and methods by which it may be implemented more widely [50].

Closely related to a culture of care is the concept of a ‘Culture of Challenge’ [51], in which decision-makers are encouraged to “choose the acceptable rather than the accepted”, and not simply repeat a procedure because “it has always been done that way”. It is critically important that every potential animal experiment is only undertaken following reflection and fullest possible application of the Three Rs. We propose that the PREPARE guidelines and associated resources are one way in which this may be accomplished. Workshops on the use of the resources in PREPARE have been arranged for this purpose, in three countries so far.

Where there is no clear scientific evidence or available guidance, decision-making in the spirit of the Three Rs may be aided by applying “the Three Ss”, a concept attributed to the American biomathematician Carol Newton: Good Science, Good Sense and Good Sensibilities [52]. This principle combines evidence-based action and (where evidence is lacking) the use of critical anthropomorphism to determine the best treatment of animals in research and testing.

### 3.2. A Network of European 3R-Centres

The current global interest in the Three Rs has played a major role in fueling the changes described above, not least through the creation of national, regional or institutional 3R-centres. The Center for Alternatives to Animal Testing (CAAT) at Johns Hopkins University in Baltimore was one of the first of these, established in 1981. Some of these are offshoots of an existing research institution (e.g., the Charité 3R Centre in Berlin) or were formed, in part, to distribute research funds (e.g., the UK NC3Rs and the Danish 3R-Center). Some have websites with large numbers of 3R-resources: the Norwegian platform Norecopa, containing links to global resources [53] which have been collected for nearly 30 years, has currently over 8500 webpages (https://norecopa.no).

Recently, an informal network of European 3R-centres was formed, during the EUSAAT congress in 2018. An interactive map giving an overview of these centers was constructed by Norecopa [54]. Representatives of the Network have, since then, met twice: in Berlin in March 2019 and at EUSAAT’s congress in October 2019. This network will facilitate coordination and sharing of resources in order to maximize animal welfare impact and to reduce unnecessary duplication of effort.

The broadest possible dissemination of 3R-related information is essential if the aspiration of Russell and Burch for animal research to be as humane as possible is to be achieved. The authors of this paper speak with many groups and individuals who are involved in the care, use and regulation of animal research and recognize that an enormous amount of excellent work is being done to apply the Three Rs in many research settings. Sadly, not all this information gets published. There appears to be a separation between ‘welfare science’ and ‘experimental science’, with most scientific publications that report animal research offering relatively little space on implementation of the Three Rs and the welfare of the animals used. We and others [55] believe that this needs to change. Close collaboration between researchers and animal carers, as recommended in the PREPARE guidelines, could help facilitate this.

Scientific meetings are clearly another means of sharing this information. The Three Rs have served as the theme for many scientific meetings in recent years. Indeed, the origins of today’s widespread interest in the Three Rs can be traced to the start of a series of World Congresses on Animal Use in the Life Sciences and Alternatives, in Baltimore in 1993. Numerous other 3R-meetings and initiatives have followed, all of which contribute to better planning and reproducibility of animal research. The series of meetings focusing on severe suffering, arranged by the Royal Society for the Prevention of Cruelty to Animals (RSPCA) is a good example of these initiatives [56]. Whilst this is encouraging, we would like to see 3R initiatives and practice embedded to a greater extent in general scientific conferences, whose participants rarely attend meetings within the field of Laboratory Animal Science. Specialists in laboratory animal science, animal welfare and in vitro technologies are rarely fully aware of the opportunities within these fields which are outside their own disciplines. This yawning knowledge gap is a major bottleneck in the advancement of the Three Rs and should, in particular, concern academic researchers conducting basic research, since this is probably where the lack of crosstalk is greatest. The situation is not helped by the fact that in vitro scientists do not necessarily communicate to the laboratory animal community that their models are in fact possible replacements for animal use. The Three Rs are now part of the legislation governing animal research in many countries across the globe, so why not include information on their implementation in all presentations where animal data is shared?

## 4. Conclusions

For over 30 years, efforts to improve the planning, reproducibility and translatability of animal experiments have focused on ways to improve reporting. While certainly needed, such improvements can never improve the quality of an experiment which has already been performed. Scientists should therefore start by using planning guidelines which attend to all the Three Rs. 

Improvement on the quality of animal studies, and efforts to replace them, require attention to all the three Rs of Russell and Burch. This will help to demonstrate to scientists that the more mathematical elements of experimental design are far from the only issues dictating the validity and success of in vivo studies.

Endorsement of this overarching influence of the 3R-concept, by all the stakeholders, will not only reduce animal numbers and improve animal welfare, but also lead to more reliable and reproducible research which should improve translation of pre-clinical studies to tangible clinical benefit. It should be the special duty of all 3R-centres to encourage greater sharing and promotion of 3R-resources, including those developed by others than themselves [57].
